# The *Parrot’s Training* in the pandemic: fallacies in India’s educational response to COVID-19

**DOI:** 10.1007/s12564-022-09739-8

**Published:** 2022-01-21

**Authors:** T. K. Krishnapriya, Padma Rani

**Affiliations:** Manipal Institute of Communication, Manipal Academy of Higher Education, Manipal, Karnataka India

**Keywords:** COVID-19, Education, Tagore, Colonization, Digital education, India

## Abstract

The paper explores India's response in the face of the COVID-19 educational crisis compared to the educational thoughts and experiments of Rabindranath Tagore, Asia's first Nobel laureate and an avid educator. The context of the study is formulated from the perception that the past and present are interconnected; hence examining similarities and dissimilarities between them can reveal latent undercurrents. A comparative historical method is employed for the study to take three diverse yet interconnected periods in the Indian education system—the Tagorean/colonial period, the post-independence period, and the pandemic period—for a continuous and comprehensive evaluation. Tagore's educational experiences evidenced that the colonial system reeled under biases that alienated the natives from their nation and its operations. Despite the efforts, the post-independence period bears remnants of the colonial system. The educational response to the pandemic also exhibits specific social, cultural, and economic tensions similar to its predecessors. As the COVID-19 lockdown implementation has  forced shutters on the educational institutions, an increasing emphasis is laid on digital education without adequate engagement with the pre-existing challenges. Under this convoluted circumstance, the paper opines that colonization continues to be the underlying process propelling the education system. Thus, the Tagorean ideals of education and its indigenous ways require urgent consideration before falling back on the old system post pandemic or endorsing digital education as the next giant stride in education.

## Introduction

At the outset, it becomes imperative to draw out the context of this study as it aims to examine the educational thoughts formulated by Rabindranath Tagore a century ago in relation to India’s educational response to the COVID-19 pandemic that has currently grasped the world. Owing to the mammoth gap between their conceptions, the two can appear to be disconnected discourses on education. More importantly, the relevance of the Tagorean ideal of education in the Indian education system was limited in its implementation even before the COVID-19 period. However, as it currently stands, the Tagorean ideal of education is more relevant than ever as the country’s response to the COVID-19 pandemic seems to have presented more problems than solutions. Hence, this paper seeks to investigate the historical influences on the present education system of India while addressing the fissures embedded within it.

The exploration begins from Tagore’s ideals of education, tracking the challenges of education post Tagore and the current response by the Indian education system. The discussion section consists of a three-way comparison between the colonial education system of Tagore’s period (1861–1941), the post-independence education system (1947–2019), and the educational response to COVID-19 (2020) by drawing parallels and marking the points of convergence and divergence, if any. Therefore, this study seeks to answer the following research questions:

R1: How is India’s response to the COVID-19 pandemic shaped by the past?
R2: What are the similarities and differences between India’s educational response to the COVID-19 pandemic and Rabindranath Tagore’s education system in colonial India?

## Literature review

### The Tagorean educational disposition in the pre-independence era (1861–1941)

Rabindranath was born into the illustrious Tagore family of Colonial Bengal in the year 1861. The socio-political temperament of the period of his birth was heavily influenced by the Indian Revolt (1857–1858) and the Bengal Renaissance. O’Connell (2003) describes the prominent Tagore family as “a virtual microcosm of the Bengal Renaissance” that served as a living university for a young Tagore.

Perhaps, it is a similar astute and simulative environment that the avid educator in Tagore attempted to recreate by establishing his experimental schools in the Bolpur district of today’s Indian state of West Bengal **(**O’Connell, 2003, 2021). He dedicated forty years of his life to constructing and fulfilling his educational vision as he founded and managed three institutions: Santiniketan, Sriniketan, and Visva-Bharati (O’Connell, 2021). Santiniketan, his first school, was established in rural Bengal in the year 1901. Visva-Bharati, his university, was established in 1918. His Institute of Rural Reconstruction was established in 1922 (later renamed Sriniketan) and aimed at rural regeneration (Mukherjee, 1970).

Tagore’s period was characterized by an overwhelming thrust of the English colonial education system, which deeply concerned him. According to O’Connell (2003), Tagore’s schools, as a response to the oppressive system, sought an active and vibrant system of education embedded in the local cultural contexts of the land. Hence, Tagore’s institutions gave utmost importance to an open-ended learning system oriented toward a trial and error method, allowing room for improvements (O’Connell, 2021). They were characterized by a profound appreciation for art and culture, cosmopolitanism, a sympathetic relationship between the student and teacher, and teaching in vernacular language (Bhattacharya, 2013; DeGiulio, 2021; O’Connell, 2003; Tagore, 2003).

The initial inspiration for the conception of Tagore’s Santiniketan was drawn from the ancient system of forest hermitage. Therefore, the school was primarily rooted in its intrinsic connection to nature. This also manifested in the curriculum of Santiniketan as the subject of nature studies served as an avenue for students to formulate deeper connections with nature through activities such as documentation and preservation of various biological specimens. The students also freely revelled in the spiritual essence of nature as they roamed around the school, and the older boys sometimes traveled to the woods at night. The classes were small in size to ensure that each student received individual attention. The subject of astronomy was explored through a small telescope that allowed the children to witness a world beyond their physical grasp. The works of literature from the East and West were also given prime importance, and even the small children indulged in listening to stories in English. Sports and other physical activities were seen as integral to the development of the children, and hence, the children played a variety of games such as football, volleyball, and tennis. They also participated and won many sports competitions outside of the school premises (Lal, 1932; Pearson, 1916). Later, the students, including the girls, practiced martial arts, including jiu-jitsu (Das Gupta, 2017; Lal, 1932). They also sang, acted, and conducted plays and indulged in other artistic endeavors, such as dance and various other vocational courses (Jha, 1994; Lal, 1932). The school also housed a library with an extensive collection of manuscripts and books in multiple languages, including Arabic, Persian, French, and German. A section of the library was specially organized for the small children. The museum at the schools displayed collections of indigenous art and crafts while substantial efforts were made under the music section to preserve folk culture and songs (Lal, 1932).

Tagore’s educational thoughts, as reflected in his schools, were highly imaginative. Nonetheless, the schools’ students were often tagged as difficult or problem children (Fraser, 2019; Nussbaum, 2009). In addition, even as these schools operated as an abode of peace, the outside environment was hostile toward the establishments. Both the British and the natives were antagonized due to their differing ideological standings compared to the schools and their founder. Apart from the lack of public support, finances were scarce even as Tagore invested most of his finances on the schools (Fraser, 2019; Jha, 1994). Notably, despite the innate method of learning and nurturing Santiniketan offered, it could not wholly occupy India’s heart even during Tagore’s period. For the outside world, the school was Tagore’s “eccentric wish fulfillment” (Fraser, 2019). Nevertheless, Tagore’s thoughts and ventures offer an insightful account of educational practices.

### Challenges in the Indian education system during the post-independence era (1947–2019)

Acknowledging the colonial contribution to the Indian education system vis-à-vis the introduction of English education, Ayyar (2017) asserts that it has done “the country a great deal of good” as it helped India transition from the medieval era. While we recognize the colonial contribution to the Indian education system, it is also imperative to address the challenges outlined in the aftermath of its struggle for independence. On the dawn of independence, India confronted a battered education system that over-emphasized authoritarian methods and was deprived of cultural context, sound vocational training, and grounded elementary education (Basu, 1989). Thereupon, post-independence India doled out educational policies conforming to the pre-existing colonial system as it relied on existing infrastructures (Wadhwa & Khatak, 2020). Further, the dire circumstances, including an abysmal literacy rate—a meager 27% of men and 9% of women were literate—and a large population necessitated the establishment of numerous schools and colleges (Basu, 1989). At this juncture, policymakers needed to act swiftly to eradicate the inequalities in education and concentrate on a paradigm addressing the development challenges (Chitnis, 1993).

Hence, the Constitution of India accommodated multiple provisions to encourage and strengthen education in India. Significant focus was placed on the underprivileged, and primary education was made free and compulsory under Article 45 of the Constitution (Dash, 2004). Moreover, various commissions were set up to evaluate the state of the education system in the country. For instance, the Radhakrishan Commission (1948–1949) recommended multiple structural modifications, including placing university education within the ambit of the Central and State governments. The percipient Kothari Commission (1964) also proposed a significant overhaul in the education system and paid keen attention to primary education, work experience, and the quality of education. Further, the commission recommended allocating around 6% of the country’s Gross Domestic Product (GDP) to the education sector (Ayyar, 2017). The post-independence era witnessed attempts to fully implement Gandhi’s and Zakir Hussain’s landmark schemes of Basic Education (1937), which intended to ensure compulsory elementary education in the mother tongue with an emphasis on craftsmanship (Wahab, 2020). The first National Policy on Education (NPE) that came out in 1968 was India’s first of its kind framework for educational policy-making. In addition, the initial decades after independence witnessed an enormous expansion in the number of educational institutions and the realm of policy-making (Ayyar, 2017).

Unfortunately, such well-intentioned provisions and various NPEs (1968, 1986, 1992; National Education Policy, 2020) failed to attain complete success as, to date, the Indian education system remains plagued by multiple challenges, including some that were evidenced immediately after independence. For instance, the Basic Education scheme was never fully realized in the educational paradigm of the country. Moreover, the Kothari Commission (1964) had recommended increasing the education expenditure and public investment to 6% of the country’s GDP. On the contrary, according to the Economic Survey 2020–2021, India’s educational expenditure in proportion to its GDP witnessed an increment from 2.8% in 2014–2015 to 3.5% in 2020–21 (Ministry of Finance, 2021). Notably, the total expenditure remains inadequate compared to the recommended investment despite all the NPEs, including the latest National Education Policy (NEP) 2020, agreeing to the Kothari Commission’s recommendation. Besides, the exponential increase in the number of educational institutions affected the quality of education as newer establishments continued to be built on limited resources (Ayyar, 2017). The proliferation of low-cost private schools with few facilities adds to this challenge as they often fail to provide quality education (Cheney et al., 2006).

Currently, the education system prioritizes numbers and records over the development of actual learning. For example, the free and compulsory education for primary students pushes for a robust schools attendance record but fails to give adequate attention to parameters such as the expected learning outcomes that would ideally yield positive results for learners (Cheney et al., 2006). Higher education is also becoming increasingly expensive, intensifying the gap between the underprivileged and privileged (Singh, 2016). In addition, a dearth of infrastructure aggravates the lack of quality education (Patel, 2013). The education system remains overwhelmed by the nation’s diversity as it still struggles to meet regional and cultural expectations. Moreover, despite the fact that the Right to Education (RTE) Act (2009) allows relaxation in the educational qualifications for appointing teachers if there is a dearth of teachers possessing minimum qualifications in a state (Ayyar, 2017), there is still a shortage of trained faculty and teachers in the education sector (Singh, 2016).

These changes and challenges clearly imply that Tagore’s educational thoughts did not translate into India’s course of education. Even though Tagore’s educational experiments influenced the Kothari Commission’s recommendations, the implications remained limited (Jha, 1994). Perhaps, the reason for this restricted contemplation of Tagore’s ideas was that it relied on a vision that the man himself could only realize. According to Nita Kumar (2015), the transmissibility and “realizable and replicable” larger practical application of Tagore’s thoughts are impeded by the singularity of his vision. Further, his educational experiments may not have suited the needs of the post-independence Indians as education was always viewed as a means to economic success (Nussbaum, 2009). Therefore, even as Tagore attempted to generate viable employment in rural areas, the culmination of his thoughts aimed to achieve the overall development of the child rather than resounding financial success.

It was amid this educational crisis and predicament that COVID-19 made its landfall in India. This has sent educational institutions into frenzy as they were suddenly forced to break the traditional classroom model of education to find a sustainable alternative.

### India’s educational response to COVID-19 (2020)

It would not be farfetched to say that the world was least prepared for the COVID-19 pandemic. When the pandemic descended upon humankind, the education sector received a severe blowback as lockdowns were implemented across the world. Students were forced to stay indoors, and almost all schools and colleges were pushed into looking for a technological alternative to replace classroom education in conducting day-to-day classes (Jena, 2020; Koul & Bapat, 2020). During the peak period of the lockdown (April 2020), 91% of students did not attend school all over the world (UNESCO, 2020).

In India, the first case of COVID-19 was reported on 30th January 2020, and a nationwide lockdown was imposed on 23rd March 2020. As a result, all educational institutions were shut, and all educational activities, including entrance exams, were put on hold. Around 320 million students in India were affected by the sudden lockdown. Despite this setback, the education sector managed to stay afloat by adopting technological measures (Jena, 2020). India employed multiple mediums—television, radio, and online apparatuses—to conduct its academic activities (UNESCO, 2020). In the case of online dissemination, Google Meet, Skype, and many other video conferencing tools were primarily used. Various platforms, such as Whatsapp, Telegram, Google Drive, and even Twitter, were also avidly used by students and teachers in higher education to share and collect information (Jena, 2020).

Even though the alternative digital education helped mimic the functioning of a traditional education system, it triggered an over-reliance on technology (UNESCO, 2020). Online education eliminates the spontaneity of face-to-face interactions and impedes the exchange of ideas between students and teachers. It, therefore, disrupted the behavioral development of children as elements such as discipline receive less scope in the process, and socialization comes to a stand-still without the physical presence of teachers and students (Wadhwa & Khatak, 2020). Moreover, online education has also spurred the question of accessibility and affordability (Rashid & Yadav, 2020). Although Indian educational institutions have resumed classes, an unfortunate ordeal remains—only 450 million of the country’s entire population has access to e-learning facilities. This is especially worrisome for students who do not have access to the basic infrastructure for online education (Jena, 2020). According to a survey published by Economic Times (cited in Raj & Khare, 2020), 1 in 4 students could not access online classes as they did not own laptops, desktops, or tablets. The Global Education Monitoring report also revealed that COVID-19 had increased educational inequalities worldwide (UNESCO, 2020). The economic divide can exacerbate the challenge of digital education adoption in the many low-income colleges and schools that operate within the country (Bokde et al., 2020; Choudhary, 2020). The feasibility and adoption of digital technology in such institutions may not be financially viable and, hence, can impede the acquisition of the necessary resources and infrastructures limiting the accessibility to digital learning.

In these circumstances, India needs to aim at providing quality education with an increased concentration on students’ overall progress to negate the impact of COVID-19 on the education sector. Therefore, a major socio-economic reckoning in the education system with an emphasis on skills, capability enhancement, and practical knowledge can be beneficial in countering the challenges brought about by COVID-19 (Bokde et al., 2020).

## Methodology

A comparative historical research methodology was employed to establish the similarities and differences of two distant periods in history, which in reference to this study, include colonial India and the current COVID-19 impaired India. Evidence on the significant developments of the periods was gathered by purposively choosing certain units for the study. Rabindranath Tagore’s writings were chosen as the primary source material on colonial India as he is an educationist who wrote comprehensively about the colonial period’s prevalent education system and devised innovative educational methods through his schools. Specific government guidelines as well as government, intra-government, and institutional circulars, independent surveys, and studies by non-governmental organizations and intergovernmental organizations were chosen as primary source materials to study India’s educational response to the COVID-19 pandemic.

Schutt (2006) recommends that a comparative historical approach should consider a sequence of events rather than just two distant events to produce wholesome results. Hence, the critical education developments post independence were traced using specific government guidelines and circulars as primary sources. The primary materials were analyzed through close textual analysis and content analysis (Lange, 2012) to produce the evidence that is presented and deliberated in the discussion section.

## Discussion

The impact of COVID-19 on the Indian education system should not be dismissed as a one-off transient phase. The pandemic should be utilized as an opportunity for a systemic implementation of a holistic education system that aims to achieve the overall, well-balanced development of a student. For the first time since the implementation of the modern education system, the displacement of classroom education has provided a key leeway for the existence of an alternate model. Unfortunately, there is little consideration for this particular aspect as India’s response to the COVID-19 pandemic seems to be rooted in two very specific outlooks. First, as lockdown rules are slowly being relaxed, schools and colleges have begun implementing offline–online mode of education. This is a clear indication that as soon as normalcy is in place, education would return to the traditional classroom model or, at the very least, to a blended model of learning. Second, there is an over-emphasis on digital education as the “new” wholesome method of education with a blatant disregard toward its internal workings and challenges. Therefore, there is a pressing need to address the challenges arising from these paradigms while pinning down the undercurrents that have shaped the current educational response to COVID-19. While addressing these, this study draws from the past to acknowledge the amendments that could result in a better future.

The discussion starts from the locus of a young Rabindranath Tagore’s educational experiences marred by the formal colonial education system and the alienation it created within the country (Mukherjee, 1970; O’Connell, 2003). A young home-bound Tagore initially considered school as a gateway to the outer world and desperately sought to join one. However, as he pursued this dream, he was met with a “resounding slap” and “sound advice” from his tutor, which he would remember a good five decades later —“You're crying to go to school now, you'll have to cry a lot more to be let off later on” (Tagore, 1917, p. 3). Initially, Tagore had attended the Oriental Seminary, and he despised the format of education he experienced as he says, “What I learnt there I have no idea, but one of its methods of punishment I still bear in mind” (Tagore, 1917, p. 3). His teachers displayed “impatience, short temper, partiality, and injustice,” which he unintentionally assimilated (p. 30). Therefore, for a young Tagore, the beginning of his school years served as a course on disillusionment with the education system. So, after his short stints at various schools, he had outright rejected all kinds of the formal education system (O’Connell, 2003). Tagore’s educational thoughts and practices are reflective of this awakening that saw him deviate away from an institutional model of education to initially implement a system based on the Indian style of hermitage schools or *tapovana*. Further, his schools sought to reform the premise and process of learning.

In Tagore’s period, the teachers were unprepared to nurture and instead chose to be imposing as most of them were thrust into an alien civilization. In his experiences, his teachers offered him less “matter” and more ill “manner.” Hence, in Santiniketan, the system was tailored to be fully different from the existing paradigm. Initially, Tagore persuaded five teachers to join Santiniketan—of which three were Christians (Fraser, 2019; Jha, 1994). Since these teachers belonged to diverse backgrounds, they brought different schools of thought to Santiniketan. Furthermore, Tagore’s schools actively strived to create an environment of intimacy between the teachers and students, and the younger teachers were lovingly addressed as *dada or didi* (brother or sister). This replaced the usual authoritarianism of the colonial system of education. In addition, the teachers themselves were not subjected to the authority of a superior; they were free to devise and implement teaching methods that they believed were in the students’ best interests. They also coordinated and managed the day-to-day functions of the school (Pearson, 1916). This way, the innovative method offered freedom of thoughts and actions to the teachers.

Post independence, the issue of employing qualified teachers plagued India. Unfortunately, the lapse of 80 years since Tagore’s death has not sufficiently bridged the dearth of adequately qualified teachers. Efforts were made in the NPE of 1968 and 1986 to address the importance of teacher training, but the legislation that followed the NPEs clearly shows that the problems continue to persist. For instance, the RTE from 2009 was amended in 2017 to grant under qualified teachers additional time to acquire the required qualifications. These ratifications indicate the prevalent problem of low-quality teaching. Besides this institutional challenge, teaching has become a demanding occupation that has pushed teachers to burn out (Shukla & Trivedi, 2008). Arguably, the teachers’ level of mental fitness and attitudes can impact the process of education and, consequently, the students (Shukla & Trivedi, 2008; Singh & Sarkar, 2015). Therefore, it is also imperative to acknowledge the challenges faced by the teachers to construct a well-developed education system.

The current educational response, which aims at combating COVID-19, has pushed teachers of various age groups and living conditions to expand their boundaries and adapt to the digital education model. A “one-size-fits-all” mechanism cannot be an expectation of technology adoption in a diverse country like India, which staggers under innumerable inequalities and intersections—in relation to economy, class, culture, gender, and problematic caste. As digital education became the norm, teachers were forced to pick up technical skills and assemble resources to conduct these classes. The new system allows very little space for the welfare of teachers as the numerous challenges it brings along exacerbate the plight of an already burned out workforce (Shukla & Trivedi, 2008). While teaching online, 63.1% of teachers lacked work satisfaction and 76.3% were worried that students were not serious about their online classes (Nambiar, 2020). Alarmingly, 42.1% of teachers agreed that they felt no motivation to teach online (Nambiar, 2020). A survey conducted in the states of Odisha, Bihar, Chhattisgarh, and Uttar Pradesh revealed that 84% of the teachers grappled with the sudden implementation of the digital education system (Vyas, 2020). The existence of a robust education system is impossible without cooperation from teachers. Therefore, the difficulties faced by teachers in the system of digital education require immediate attention.

Tagore had once said, “he who has lost the child in himself is absolutely unfit for this great work of educating human children” (Tagore, 1947a, p. 5). Following this, it may be accurate to posit that some of the problems mentioned above can be solved by the teachers’ creative negotiation and engagement and efforts to perceive the problems through students’ eyes. However, the imaginative involvement from the teachers cannot be the exclusive solution to the current problems, and adequate redressal must be meted out by the authorities to handle the COVID-19 induced burden and technological anxieties. Nonetheless, Tagore’s educational experiences with the colonial system converge with the currently deployed response to COVID-19 as teachers in both scenarios appear ill-prepared to meet the requirements for the overall development of their students.

Moving forward, Tagore was frustrated with the colonial education as it mainly aimed at the production of a working class for fulfilling the job requirements of the British administration. He yearned for a system of education that was not mass-produced but individualistic in its scope. In his article *Purvaprasner Anubritti,* he opines that:

If we place education in the hands of the government, they will attempt through that education to fulfil their own interests and not ours… they will not bother to make him a true citizen of India. We can impart education according to our desire only if we take education in our hands (Tagore as quoted in Jalan, 1976, p. 10).

He staunchly protested the exam-centric nature of the education system, which aided in the mass production of “the educated” and “refused to pin his faith on examinations” as he believed the ultimate goal of teaching was not to shout out meanings but to initiate the young minds to endless possibilities of the world and the beyond. A student writing essay paragraphs cannot contain such an awakening as the experiences are stronger than what he can write or fathom (Tagore, 1917). In Santiniketan, lower classes were not allowed to take examinations—save for an annual exam that teachers would administer to evaluate the overall progress of each individual child. Further, students in higher classes sat in an environment of their choice while doing the examination as the mutual trust between the students and teachers was considered to be of paramount importance. As Pearson (1916) says, sometimes “trust begets trust,” which probably worked in favor of such unusual methods (p. 56).

In post-independence India, glimpses of the exam-centric education system were evidenced in multiple legislations. This stifled the creative and individualistic progress of the students. However, the first NPE sought to make examination secondary compared to the comprehensive and complete development of a student. It stipulates that:

evaluation [should] be a continuous process aimed at helping the student to improve his level of achievement rather than at ‘certifying’ the quality of his performance at a given moment of time (NPE, 1968, p. 42).

However, this was soon undone as the NPE, 1986 envisaged (and later implemented) a standard entrance test for admission to higher studies after the 12th grade. Similar tests were also considered for admission to a master’s degree. This essentially rolled back the first NPE and its vision while unveiling an exam-centric education system.

A push for a similar exam-centric standpoint in place of a creative and experimental learning method is also evident in the COVID-19 educational approach. As digital learning became the primary tool for education, proctored examinations became the norm (India Today, 2020; Times of India, 2020). Thus, the narrative surrounding the education system in India remains as exam-centric as it used to be, throttling any scope for betterment. A press release by the University Grants Commission (UGC) (dated July 18, 2020)—a statutory body tasked with managing higher education India—on conducting examinations during the pandemic says:

… examinations are an integral part of the education system and a measure of students’ learning, knowledge, skills, and other competencies. (UGC, 2020, p. 1).

As exams were postponed due to the lockdown and the subsequent closure of educational institutions, the circular directed universities to hold terminal examinations to “safeguard the larger interests of the students” at a global level. Data obtained by UGC from 755 (out of a total of 945) universities across India revealed that 560 of them had either held the examinations during the pandemic or were at various stages of administering them (UGC, 2020). All India Council for Technical Education (AICTE)—a statutory body tasked with managing technical higher education—directed its affiliated universities and institutions to follow the guidelines provided by UGC in notifications dated April 15, 2020 and May 01, 2020﻿ (AICTE, 2020a, 2020b). Unfortunately, this system offers no space for creativity and self-actualization. Instead, it augments the practice of creating a mass workforce, which works in favor of the government and its beneficiaries—a disposition similar to Tagore’s colonial India. Lim (2010, p. 157) opines that “the exam-centric Asian education system has created a workforce more adept at imitation than innovation,” and the COVID-19 response does not seem to provide a respite from this.

Tagore’s Santiniketan actively moved away from the mindless bookish learning. The method was deemed to be devoid of intrinsic prospects of child-centric education oriented toward a system of learning stemming from the students’ efforts as their curious minds explored the world around them. Further, Tagore made an excellent commentary on the shortcomings of the educational system of the colonial period in his short story *Tota* *Kahini* (1944) or *The* *Parrot’s Training* (1944). The story spoke of a bird—ignorant and ill-mannered as “it sang all night, but never recited scriptures. It hopped pretty frequently, but lacked manners” (p. 10). The King of the land decided that the bird should not stay ignorant, and the pundits declared that the “ignorance” of the bird was due to its “natural habit of living in poor nests” (p. 13). They, therefore, decided that a “suitable golden cage” would be appropriate in educating the bird (p. 13). They made texts by copying from books and copying from copies till the pile of material touched the sky. The cage was well polished and maintained, while the bird was fed the leaves of the manuscripts. The world around the cage flourished except for the bird within the cage. The King visited the “Department of Education’s Hall of Learning” and established that the method of teaching was so “stupendous that the bird looked ridiculously unimportant in comparison” (p. 22). However, as the choked bird fluttered its wings, it was chained and further taught by pundits with a “text-book in one hand and baton in the other” (p. 25). The bird passed away, but that went unnoticed, and when poked, the bird did not fly or hop; only the stuffed manuscripts in its body swished. Hence, the King declared that the bird’s education was complete.

The story was a scathing take on the education system, which removed one from their natural surroundings and innate workings to chain them to classrooms where knowledge is force fed. As pointed out before, the colonial education system did not aim at the overall development of a student—this explains why Santiniketan opted to create an education system free of rote learning methods. Unfortunately, the post-independence system fell into the trap of memorization. Similarly, the COVID-19 educational approach has already adopted the time-tested classroom education method based on “scriptures.” For example, a circular released by the Indian Institute of Technology (IIT) Bombay (2020), one of India’s most prestigious institutions, on the conduction of online classes emphasized 20 h of classroom learning for its students. Various e-learning portals of India have simply shifted from physical classrooms to virtual classrooms but maintained the same model of education. Therefore, it would not be farfetched to say that COVID-19 has managed to transform the golden cage of institutional education into a more embellished allegorical golden cage of digital education.

The method of a mass-directed and authoritarian classroom education is deeply embedded in the system to an extent that the first and foremost reaction to COVID-19 was to recreate a similar environment in a virtual world free of the coronavirus. According to a governmental report from Ministry of Education (2020)  titled “Indian National Commission for Cooperation with UNESCO Response to COVID-19”, India’s Ministry of Human Resources Development’s (MHRD) response to COVID-19 immediately after the lockdown was to “intensify digital learning with equity.” Various digital platforms, reliant on laptops, computers, and mobile phones, were used for this purpose, accompanied by televisions and radios for remote areas. It therefore appears that the immediate solution to the disruption of classroom education triggered by the COVID-19 crisis was solely perceived to be technological interventions with ethos dating back to colonial times.

As technology is deemed the way forward for the world, reliance on technology to a certain extent may be considered a requirement. However, there is an evident over-reliance on technology in the form of virtual classrooms and proctored examinations that hinder the students from exploring their interests. In addition, this crisis is further exacerbated by the methods adopted in online teaching. Since offline classes were merely “shifted” online, concerns as to whether appropriate online teaching methods were adopted cannot be ignored. Online teaching requires alternative approaches to teaching rather than the traditional classroom education, especially in the case of young children (Dong et al., 2020; Hurlbut, 2018; Sathish et al., 2020). For instance, various states in India, such as Kerala, started televised classes called “First Bell” for school-going pupils. However, the idea of mass production of education is not adequate enough to meet the needs of individual students, especially the younger lot. In the case of younger children, Tagore opines that:

The school master is of opinion that the best means of educating a child is by concentration of mind, but Mother Nature knows that the best way is by dispersion of mind … constant occasions to explore one’s capacities through surprises of achievement (Tagore, 1947a, p. 4).

Thus, recorded lectures without actual stimulation of minds are equivalent to the manuscripts the Parrot was forced to gulp down. As in the story, the “stupendous” method of digital education has overshadowed the importance of the students in the education system as the COVID-19 response is solely being shaped around the narrative of technology.

Moreover, a significant concern for any education system in a developing nation would be its availability and affordability to an ordinary person. For Tagore, the divide between the have and have-nots was “impassable,” and he thought that villages, which are the cradles of culture, remained utterly marginalized in the larger picture:

The villagers have neither education, nor medical care, nor do they possess wealth, food, and clothing. On the other side, those who read in the college, practice law or medicine or pile up money at the banks find themselves on an island surrounded by bottomless separation (Tagore, 1957, p. 26).

Tagore’s various educational ventures, such as Sriniketan, reflected his criticism against the impermeability of modern pursuits in the lives of the rural poor. The school was primarily aimed at rural reconstruction. Activities at the school involved farming, poultry management, weaving, pottery, various small industries, tailoring, scout organizations, and other educational and vocational ventures tailored to rural living and upliftment (Jha, 1994; Lal, 1932). Further, students were required to take up a hands-on trading activity, and handicraft was a key area of concentration.

The post-independence government of India adopted the program at Sriniketan in its five-year plan as a feasible approach to rural development (Jha, 1994). Furthermore, the post-independence period witnessed various other governmental attempts to make education accessible to the needy. Free and compulsory education was part of NPE 1968. It also extensively dealt with education in rural areas, with particular emphasis on quality education. However, these efforts did not reach complete fruition as the education system failed to bridge the massive gap between the rich and the poor.

An Oxfam India report states that it would take 941 years for a minimum wage worker in India to earn as much as a year’s salary of a high paid employee in an Indian garment factory (Himanshu, 2019). In addition, the unaffordability of private education and the increasing expenditure of higher education aggravate the existing inequalities as it denies education to the poor. This inequality is more alarming than ever as half of India’s population is under the age of 25, and it is imperative to provide them with adequate education for their personal betterment and successful nation-building. According to UNICEF data (n.d, retrieved on January 26, 2020), there are specific fissures and intersections visible in the matter of educational disparity. Out of the six million children away from schools, the most belong to marginalized communities, and they are primarily concentrated in the northern parts of India. Hence, the cycle of inequality drives the poor further away from the opportunities of education and consequently pushes them deeper into the clutches of poverty.

The realities of the economic and social inequalities have transcended to the digital age. Even as India touts 600 million internet users, almost half of India’s population lacks access to the internet. These numbers are even lower in rural areas, as only 32% have internet access (Beniwal, 2020). Moreover, device usage and ownership are impeded by social and economic inequalities. For instance, internet and digital device use vary across genders as Indian women are largely marginalized. A low-income household in India needs to spend 3% of their monthly income to afford one gigabyte (GB) of data (Nikore & Uppadhayay, 2021).

As the COVID-19 educational approach adopts the motto of “digital,” the brunt of the unsustainable method would be borne by the economically and digitally deprived. Therefore, the effectiveness of deploying digital education as a response to COVID-19 is questionable. Even as MHRD attempted to make digital education equitable by using radio and television as mediums of disseminating knowledge, its feasibility is limited. Such a mass-directed education approach disregards the students’ varying abilities and fails to account for the cultural, linguistic, geographical, and economic contexts. According to IIT Bombay’s (2020) circular on conducting exams, the students are required to have a laptop with a webcam, which was the norm for most proctored exams, or two mobile devices. This might be impossible to amass for students from low-income rural families in a country where only 450 million people have access to e-learning facilities (Jena, 2020). Unfortunately, the state of Kerala reported India’s first student suicide on 2nd June 2020 as the child could not access online/televised classes. Kerala’s education department had rolled out televised classes (First Bell) from June 01, 2020, which the student could not access due to a broken television set (Lathabhavan & Griffiths, 2020; NDTV, 2020). Further, a status report from Oxfam India indicates that 80% of parents reported that their children did not receive education during the lockdown (Vyas, 2020).

Hence, the rich/poor and urban/rural dichotomies manifesting in the education system do not present a brighter picture compared to the past. Further, the integration of technology into the existing disparities has amplified the economic and social divide. Perhaps, it is important to look at Tagore’s educational experiments as a manifestation of an in-depth understanding of the varying socio-economic circumstances of children as the schools refused to adopt a resource-centric method of learning that would be biased toward the rich. In the context of Tagore’s schools, Jha (1994) says that an education system solely dependent on books was unsuitable for ordinary people in the colonial period. As digital resources become the new essential for education, the extent of their viability in a developing world needs immediate introspection.

Furthering the discussion, Tagore pointed out that a significant contributor to the nation's educational impoverishment was the non-usage of mother tongues/regional languages in teaching. He felt that the modern education system had been dragged out of its native element and culture. The elite education system in India was surrounded by “a thick wall of an alien tongue” (Tagore, 1947b, p.16). During Tagore’s period, elementary education was taught in mother tongue. Macaulay’s infamous treatise was an interesting and yet unfortunate culmination in the history of British Education policy; it required the use of English as the medium of education in Indian institutions henceforth (Cutts, 1953). Apart from a blatant disregard for the intellectual capability of Indian students, Macaulay’s discourse assured the alienation of the colonized from their own education system. The minute exhibited certain contempt toward the native culture (Macaulay, 1835). For Macaulay, the colonized languages or books were of no comparison to the British’s:

medical doctrines which would disgrace an English farrier, astronomy which would move laughter in girls at an English boarding school, history abounding with kings thirty feet high and reigns thirty thousand years long, and geography made of seas of treacle and seas of butter (Macaulay, 1835, p. 4).

Tagore was in awe of Japan, where modern knowledge was taught using one’s mother tongue. He believed that Japan’s realization of the importance of mother tongue stemmed from the need to ensure that people from all classes had unrestricted access to education. On the imposition of English education, Tagore opined that:

To say that adequate pursuit of education is not possible except through the English language is the same thing as saying that nourishing food cannot be had save from the manager of an English hotel (Tagore, 1947b, p. 27).

In Tagore’s schools, Bengali was the primary language and was foremost taught to the children. They read all kinds of literature in Bengali. Once the children mastered the mother tongue, basic English verbs were introduced. This was then followed by teaching the rest of the verbs through dramatization. Then a “comparative method” of translation from English to Bengali was employed and gradually replaced with translation from Bengali to English, thus enabling an enhanced understanding of the structure and semantics of both languages (Kumar, 2015). At the same time, it placed much stress on the value of regional languages.

Tagore was vehemently against the idea of English teaching, which prevented the ordinary from attaining education (Tagore, 1947b). Further, Santiniketan exemplifies an innovative comparative method of language learning, which is perfectly suited for a country such as India with 121 languages and 270 mother tongues (Census of India, 2011a). Even though post-independence India has made efforts to include regional languages in the education system (NPE, 1968), students generally deal with English at the tertiary education level. Ramanathan (2012) highlights the intersection of caste and class divide in learning English.

The COVID-19 response to education also faces a similar challenge as English is the main language used in various digital interventions such as applications and software. A﻿﻿c﻿cording to the Census of India (2011b), English is the primary language of only 2.56 million Indians. Thus, the language skills required to utilize technology can pose a barrier to digital education adoption. Also, most of the digital content platforms (including the government regulated ones) have English as their primary language. This can be a challenge in the diverse Indian scenarios.

The use of English as a primary language that began with implementing the modern education system created a platform that alienated Indians from understanding the pulse of their rural areas while providing them with ample opportunities to understand and accept Western thoughts and literature (Tagore, 1947b). Unfortunately, a similar alienation continues to exist in the country as the COVID-19 propelled education system does not adequately address and integrate the people of all linguistic, class, caste, gender, and socio-economic backgrounds.

Finally, this study opines that the COVID-19 educational response appears to bear a latent colonial undercurrent in its trajectory. In this context, it is imperative to remember that colonization does not merely colonize a land but also the minds and history of the victims (Fanon, 1961). The colonial hangover is not only evidenced in the obvious use of the English language, but parallels can also be drawn between the teacher–student relationships. During the colonial period, the alienation of teachers from students was embedded in the modern education system as teachers were thrown into a foreign culture, for which they often held contempt. In the COVID-19 scenario, the alien culture is replaced by the alien mode of education—digital technology. Both barriers forbid a real connection between teachers and students. Further, multiple aspects of colonial times were evidenced in the post-independence Indian education system: the negligence toward the indigenous ideologies/language while looking up to and absorbing from the West; the restricted education of the poor and the preference for the elite; lack of individualistic attention on the students; ignorance of the plight of underprivileged; implementing rote learning methods; and mass production of education through an over-reliance on exams and classrooms. Unfortunately, the educational response to the current COVID-19 is not bereft of these features. The digital education system is borrowed from Western technology, exam-centric rote learning is still considered an efficient method, lectures are produced for mass consumption, the education system caters for the elite who can afford the best of the technology, and the over-reliance on classroom education has simply been overtaken by digital classrooms.

Therefore, the latent temperament that shaped the current response to COVID-19 is colonization and the model of education set up during the period. As a result, a deeper introspection is required before endorsing digital technology as the subsequent break-out development in education. The juncture appears more crucial since the implementation of the National Education Policy 2020, which seeks significant refurbishment of the current education system, has already been doled out.

## Conclusion

COVID-19 has had an immense impact on the education system of India. Even though the government deployed a carefully curated response, certain inconsistencies and inadequacies highlighted in this study require immediate redressal. On the one hand, multicultural India cannot follow the Western method of digital-centric education as technological, social, and economic inequalities can negate the benefits of the cause. This may tamper with all the progress the education system has so far achieved in facilitating equal access to education. On the other hand, as COVID-19 shows signs of receding with the induction of immunization programs, the education system may turn lethargic and fall back on its tried and tested formula of the colonial method of education. This will obstruct the current discussions and debates that can act as a catalyst for necessary changes and implementation of NEP 2020. NEP 2020 aims to restructure the education system by incorporating flexible courses with an enthusiastic inclusion of arts into education. However, the implementation process is dependent on multiple government bodies (NEP, 2020).

Hence, this study opines that the crisis propelled by the pandemic must be exploited—but not just negated or obliterated—to eradicate the underlying colonialism still persistent within the Indian education system. This will be instrumental in questioning the viability of both the new digital education system and the old colonial education system.

A complete overhauling of the education system may not be the way of progression in India due to its high population and inadequate infrastructure. However, considering this as a point of transgression, a reconstruction of education appears relatively easy as COVID-19 has driven students out of schools and colleges. A few areas of deliberation for such a reconstruction can entail emphasizing the holistic development of students; educational institutions amending their cage-like structure meant for mass production of the “educated” working class; the inclusion of arts; the importance placed on mother tongue/regional language; paying attention to the rural poor; boosting India's cultural standing; withdrawing rote learning; and the thrust on cosmopolitanism. Moreover, as the reliance on technology shows no signs of decline worldwide, India would need to make particular strides in this direction. In that case, the Indian system needs to streamline the methods of imparting education through technological platforms. The thrust must center on removing social, economic, and cultural barriers, and the medium must not engulf the method of education; instead, a balance must be established between the purpose of education and the medium of education. Tagore rightly points at the necessity of such a balance as he speaks about the aim of education:

We have come to this world to accept it, not merely to know it. We may become powerful by knowledge, but we attain fullness by sympathy. The highest education is that which does not merely give us information, but makes our life in harmony with all existence (Tagore, 1947a, p. 1).

Further, even as the above suggestions fall in line with Tagore’s vision for a comprehensive education system, a detailed, multifaceted examination of the best “new” educational approaches (derived from a Tagorean point of view) that can be employed in the post-pandemic world is beyond the scope of the current study. Hence this study did not explore the possible educational strategies that India can pursue hereafter based on a Tagorean model. Nevertheless, Tagore’s educational thoughts and implementation offer a differing perspective from the customarily accepted models of education evidenced in the historical and present India.

## Data Availability

Not applicable.
